# Simulated weightlessness procedure, head-down bed rest impairs adult neurogenesis in the hippocampus of rhesus macaque

**DOI:** 10.1186/s13041-019-0459-y

**Published:** 2019-05-09

**Authors:** Xu Zhang, Xixia Chu, Lei Chen, Juan Fu, Shuai Wang, Jinjing Song, Guanghan Kan, Weizhong Jiang, Guang He, Xiaoping Chen, Weidong Li

**Affiliations:** 1 Bio-X Institutes, Key Laboratory for the Genetics of Development and Neuropsychiatric Disorders (Ministry of Education), Shanghai Key Laboratory of Psychotic Disorders, and Brain Science and Technology Research Center, Institute of Psychology and Behavioral Sciences, Shanghai Jiao Tong University, 800 Dongchuan Road, Shanghai, 200240 China; 2 Department of Obstetrics and Gynecology, the First Affiliated Hospital of Dalian Medical University, Dalian, China; 3 National Key Laboratory of Human Factors Engineering, China Astronaut Research and Training Center, Beijing, 100094 China; 4 Department of Neurosurgery, Minhang Hospital, Fudan University, Shanghai, China

**Keywords:** Adult neurogenesis, Simulated weightlessness, Rhesus monkey

## Abstract

The microgravity environment in space can impact astronauts’ cognitive and behavioral activities. However, due to the limitations of research conditions, studies of biological changes in the primate brain, such as neurogenesis, have been comparatively few. We take advantage of − 6° head-down bed rest (HDBR), one of the most implemented space analogue on the ground, to investigate the effects of weightlessness on neurogenesis of non-human primate brain. Rhesus Macaque monkeys were subjected to HDBR for 42 days to simulate weightlessness. BrdU (5-bromodeoxyuridin) and IdU (iododeoxyuridine) were intraperitoneally injected separately before or after HDBR to label the survival and proliferation of newborn neurons. Immunohistochemistry was performed to study the effect of simulated weightlessness on neurogenesis. BrdU staining showed that survival of newborn neurons was reduced, while there were fewer BrdU-positive neurons in the HDBR group compared with the control. Furthermore, IdU-positive neurons also decreased in the HDBR group suggesting a reduced proliferation capacity for these newborn neurons. Our results demonstrate the definite neurogenesis in the adult rhesus macaque hippocampus, and simulated weightlessness HDBR procedure impairs the adult neurogenesis.

**Electronic supplementary material:**

The online version of this article (10.1186/s13041-019-0459-y) contains supplementary material, which is available to authorized users.

There have been many long-duration spaceflights over the past decades, and more spaceflights with even longer durations will be required in the future. Humans, instead of robots, have an essential role in long-duration spaceflight missions due to superior perception, intelligent decision-making and capacity for independent action. It is clear that the microgravity environment in space can impact astronauts’ cognitive and behavioral activities [1, 2], which further affects the astronauts’ decision-making [3]. This could be noteworthy risk for long-duration spaceflight missions. Therefore, it is of great importance to reveal the underlying mechanism of how microgravity leads to abnormal cognitive and behavioral activities. Physiology studies reveal that changes in volumes of cerebrospinal fluid, cerebral blood flow and intracranial pressure are caused by the redistribution of an astronaut’s body fluid toward the head in a weightless environment [4, 5], and this may lead to structure remodeling. Neuroimaging studies have demonstrated alterations in the volumes of gray matter and white matter in specific brain regions including the frontal lobes and the hippocampus [6]. Early studies have also shown microgravity affects neurotransmitter concentrations [7] and the number of synapses [8].

Most studies on brain tissues under microgravity have been based on rat and mouse animal models [9], which might not simulate human activities well. Previous studies in human have reported the effect of spaceflight on psychological problems, cephalic fluid shifts, and cognitive alterations, however biological changes in the brain are not as well investigated [10]. There are few reports on whether brain abnormalities caused by space flight are related to neurogenesis. In the last decade, there is increasing evidence demonstrating the important role of adult hippocampal neurogenesis in the pathogenesis and therapeutics of mental diseases. Head-down bed rest (HDBR) is the widely used procedure to study the effects of simulated weightlessness on primates on the ground. HDBR eliminates gravitational input form the head to the leg and inducing cephalic fluid shift from the lower parts of the body toward the head by applying bed rest with 6° head down position ([11], Fig. 1a and Additional file 1: Figure S1). However, the subjects are still under normal gravity during the HDBR procedure. With the advantage of HDBR animal model, we can test if simulated weightlessness could affect neurogenesis and speculate the effect of microgravity on the primate brain.

**Fig. 1:**
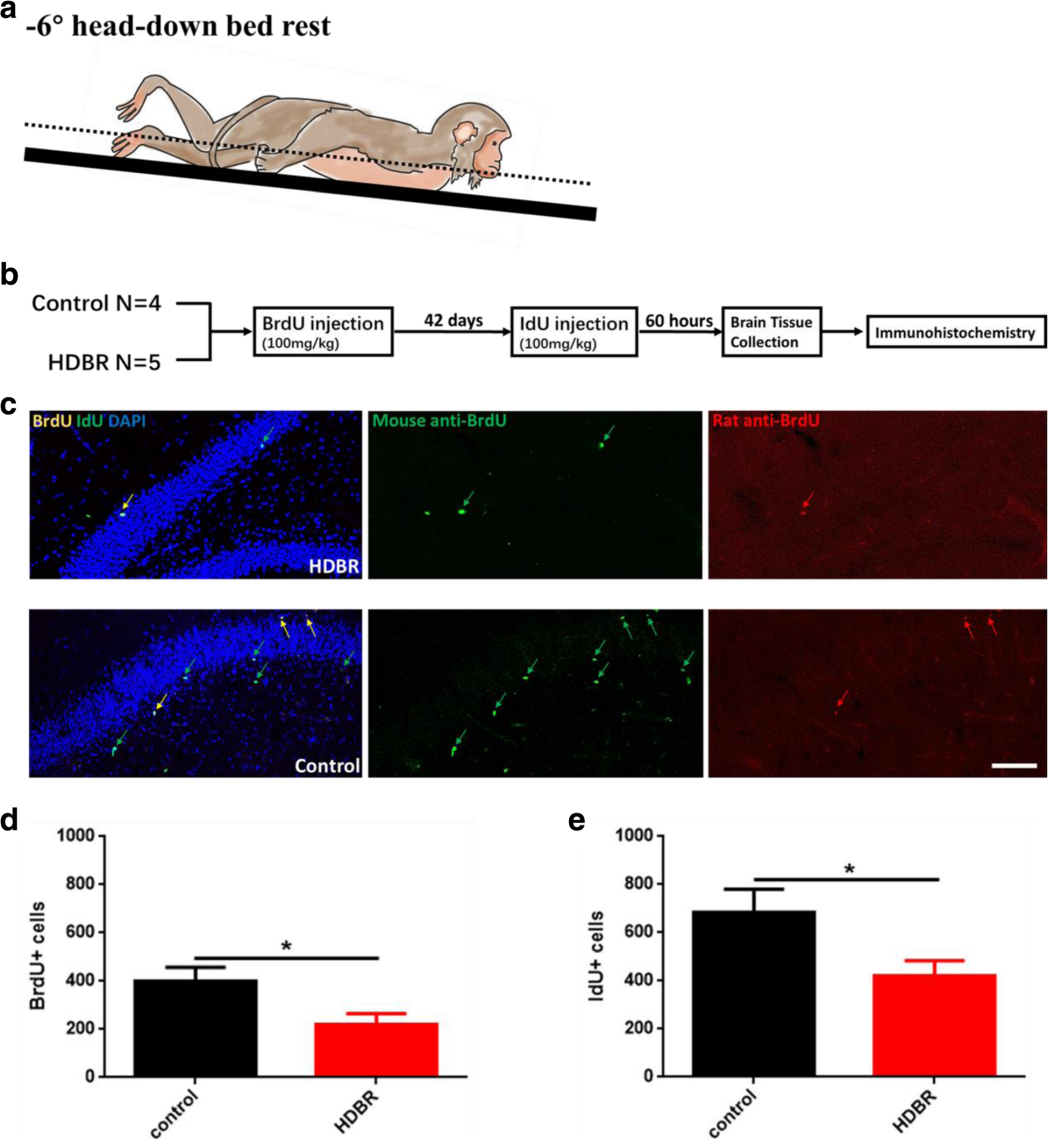
HDBR impairs neurogenesis. **a** Schematic diagram of monkey in HDBR group. **b** Experimental procedure of immunohistochemistry. **c** Representative images of BrdU and IdU staining in HDBR (above) and control (below). **d-e** Quantitative results of immunofluorescence imaging of BrdU (**d**) and IdU (**e**) in the DG of HDBR and control monkeys. Data are shown as the mean ± SEM. *P < 0.05. Scale bar, 200um

In this study, 5 monkeys were subjected to HDBR for 42 days to study the effects on the brain. Simultaneously, monkeys in the control group were single housed in the cages in the next room. While muscle atrophy and bone loss were also studied, those results are outside the scope of this paper. We collected tissue from the monkeys to examine whether HDBR for 42 days had an influence on adult hippocampal neurogenesis. Both BrdU (5-bromodeoxyuridin) and IdU (iododeoxyuridine) were intraperitoneally injected to label the newborn neurons. BrdU was injected before HDBR, while IdU was injected at the end of HDBR (Fig. 1b). Two primary antibodies, rat anti-BrdU (react with BrdU only) and mouse anti-BrdU(react with BrdU and IdU), were used for the separation of BrdU and IdU. BrdU-positive and IdU-positive cells in dentate gyrus (DG) were counted to represent the capacity of neurogenesis. We found significant reduction of cell survival by BrdU labeling (HDBR, *N* = 5, control, *N* = 3) and decreased cell proliferation by IdU labeling (HDBR, N = 5, control, *N* = 4) in HDBR group compared with the control (Fig. 1c-e and Additional file 1: Table S1). These results demonstrated that 42 days HDBR impairs adult hippocampal neurogenesis.

Hippocampal neurogenesis has been observed in different adult animals. Studies have indicated that the newly generated cells might have a function in cognition and brain repair [12]. Adult hippocampal neurogenesis is also found in humans and contributes to memory function and enhanced synaptic plasticity across the life span. Adult hippocampal neurogenesis adds particular functionality to the mammalian hippocampus and presumably is involved in cognitive functions that we consider to be essential for humans [13]. Recent research found that recruitment of young neurons to the primate hippocampus decreases rapidly during the first years of life, and neurogenesis in the DG is extremely rare in adult [14], however, our results strongly prove that adult neurogenesis was still continued in adult monkeys.

During HDBR procedure, the monkeys were restrained on the bed. Several studies showed that volunteers developed psychic stress, and the plasm hormone involved in the response of the organism to stress, such as cortisol was significantly altered in human HDBR research [11]. Restraint stress has also been related to decreased cell proliferation and survival of the newborn hippocampal granule cells in mice. [15]. The decreased neurogenesis was caused by HDBR procedure, which might contain the effects from cephalic fluid shift and stress.

In conclusion, our results indicated the unambiguous neurogenesis in the adult rhesus macaque hippocampus, and simulated weightlessness HDBR procedure impairs the adult neurogenesis.

## Additional file

Additional file 1: **Table S1.** Materials and Methods. Raw data of immunohistochemistry analysis. **Figure S1**. The Photo of monkey in HDBR group (DOCX 445 kb)

## Abbreviations

BrdU: 5-bromodeoxyuridine
DG: Dentate gyrus
HDBR: Head-down bed rest
IdU: Iododeoxyuridine
PBS: Phosphate buffered saline
PFA: Paraformaldehyde
