# Supplementary material for: Simulated weightlessness procedure, head-down bed rest impairs adult neurogenesis in the hippocampus of rhesus macaque
Source: Mol Brain. 2019 May 9;12:46. doi: 10.1186/s13041-019-0459-y (PMC6509794; doi:10.1186/s13041-019-0459-y)
Supplement: Supplementary file 1 — Table S1. Materials and Methods. Raw data of immunohistochemistry analysis. Figure S1. The Photo of monkey in HDBR group (DOCX 445 kb) [file 13041_2019_459_MOESM1_ESM.docx]

Additional file 1

**Simulated Weightlessness Procedure, Head-down Bed Rest Impairs Adult Neurogenesis in the Hippocampus of Rhesus Macaque**

Xu Zhang^1,#^, Xixia Chu^1,#^, Lei Chen^1,#^, Juan Fu^2,#^, Shuai Wang^1^, Jinjing Song^1^, Guanghan Kan^3^, Weizhong Jiang^4^, Guang He^1^, Xiaoping Chen^3,^*, Weidong Li^1,^*

^#^ These authors contributed equally to this work.

*Corresponding author. E-mail: liwd@sjtu.edu.cn; xpchen2009@163.com

**This file includes:**

Materials and Methods

Table S1

Figure S1

**Materials and Methods**

**Animal**

Nine healthy male rhesus monkeys, aged 4 to 6 years and weighing 4 to 8 kg, were purchased from Beijing Institute of Xie’erxin Biology Resource (Beijing, China). All of these monkeys received 3 months of domestication (involving preliminary caretaker handling, confinement jacket fitting, and tilt-table acclimation training) at the Laboratory Animal Center of China Astronaut Research and Training Center prior to the start of the experiments. A 6-week head-down bed rest (HDBR) experiment was performed on five monkeys to simulate weightlessness. Briefly, rhesus monkeys laid on beds, which were tilted backward 6° from the horizontal. The head-down monkeys wore the confinement jacket, which enabled them to be fixed to the bed. Since two hands were not fixed to the bed (Additional file 1: Figure S1, monkeys had free access to food and water, including fruits. The monkeys were housed one per bed in rooms with air temperature maintained at 23 ± 2°C and a standard 12:12 h dark–light cycle (lights were turned on at 8:00 a.m. and off at 8:00 p.m.). 2 to 3 HDBR monkeys were placed in each room. The size of the room is about 30m^2^, and the size of each bed is 80cm * 120cm. Monkeys in the control group were single housed in the cages in the next room. Throughout the duration of the experiment, monkeys received sufficient humanistic care, for example, toys (such as the drum-shaped rattle, a Chinese traditional toy) were available all the time. The caretaker accompanied the monkeys during the daytime to help relieve anxiety. Furthermore, caretaker checked the monkeys for defecation several times a day and cleaned them up to keep the monkeys in clean condition. The general health condition of the monkeys was also carefully monitored. All procedures were performed in accordance with the principles of the Association for Assessment and Accreditation of Laboratory Animal Care International (AAALAC), approved by Institutional Animal Care and Use Committee of China Astronaut Research and Training Center (ACC-IACUC-2014-001).

**Immunohistochemistry**

BrdU (100mg/Kg, Sigma) was injected before HDBR, while IdU (100mg/Kg, Sigma) was injected at the end of HDBR. The samples of experimental monkeys such as blood, bones, muscles, etc. were also collected by other researchers for investigating the effects of HDBR condition, so we can not use PFA (paraformaldehyde) for in vivo fixation. The monkeys were deeply anesthetized and sacrificed. Blood was extracted. Then, brains were removed from the skull, post- fixed in 4% PFA (Sigma) for 48h at 4 °C, and dehydration in 30% sucrose in PBS (Phosphate Buffered Saline) solution for 5 weeks until sunk to the bottom. Coronal brain sections were obtained by a cryostat (CM3050 S, Leica) at 40 μm and every 24 sections were collected in one well. Totally, there were 24 wells and average 15 brain slices in each well. For BrdU and IdU staining, the slices were denatured for 30 min at 37 °C with 2 M HCl, neutralized by 10 min with 0.1 M borate buffer (pH 8.5), then washed with PBS (pH 7.4) three times for 10min. Sections were blocked by 5% goat serum in PBS solution containing 0.3% TritonX-100 (PBST) in room temperature for 1h. Then the sections were incubated with the primary antibodies: rat anti-BrdU (1:500, Abcam, react with BrdU only) and mouse anti-BrdU（BD, 1:100, react with BrdU and IdU）overnight at 4 °C in a wet box. After being washed with PBS three times for 10min each, secondary antibodies, 488 anti-mouse (Green) and 594 anti-rat (Red) (Invitrogen life technologies), were reacted for 2 hours at room temperature and followed with DAPI (Sigma-Aldrich). The quantity of BrdU-positive and IdU-positive cells in DG were counted from images obtained under a Leica confocal microscope. IdU-positive cells were labeled with green color only, while BrdU-positive cells were overlapped with both green and red color.

**Statistical analysis**

P values <0.05 were considered statistically significant (*P < 0.05, ** P < 0.01, *** P < 0.001). All data were presented as means ± SEM and were analyzed using GraphPad Prism software. An unpaired two-tailed t test was used to measure the statistical differences between the two groups.

**Table S1.** Raw data of immunohistochemistry analysis

|  | **Animal ID** | **IdU** | **BrdU** | **Total IdU** | **Total BrdU** |
| --- | --- | --- | --- | --- | --- |
| control | 082285 | 16 |  | 768 |  |
| control | 081675 | 12 | 10 | 576 | 480 |
| control | 090605 | 19 | 9 | 912 | 432 |
| control | 070145 | 10 | 6 | 480 | 288 |
| HDBR | 082053 | 5 | 2 | 240 | 96 |
| HDBR | 082525 | 7 | 6 | 336 | 288 |
| HDBR | 090651 | 11 | 7 | 528 | 336 |
| HDBR | 080747 | 12 | 5 | 576 | 240 |
| HDBR | 081477 | 9 | 3 | 432 | 144 |

***Monkey 082285** in control group was injected IdU only.

*****For each monkey brain, **half** of hippocampus was separated into **24** wells. Each well consisted of 15 brain slices. All slices from one well were counted for the number of BrdU and IdU.

**Total BrdU= BrdU *2*24. Total IdU= IdU *2*24.**

**
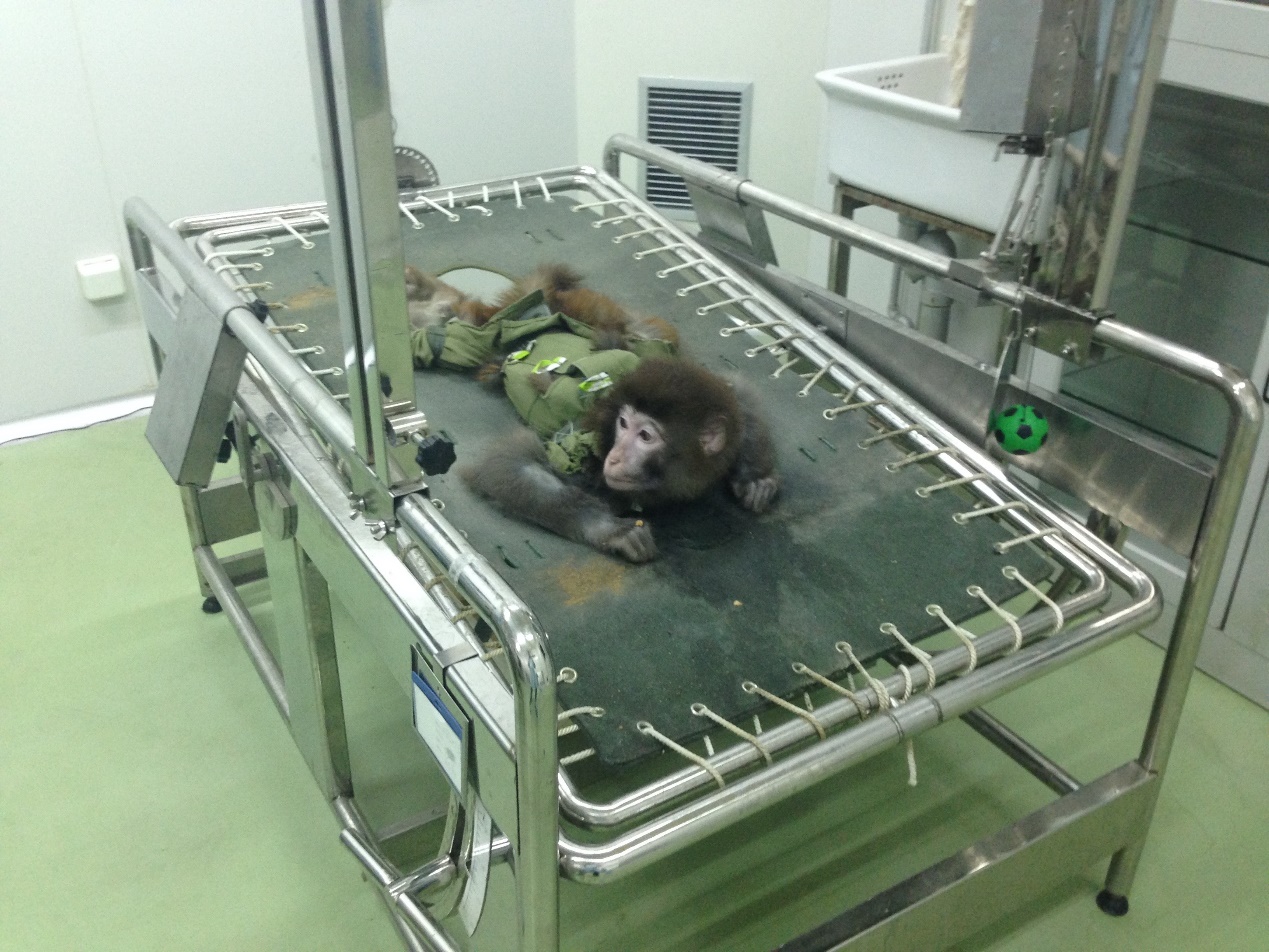
**

**Figure S1.** The photo of monkey in HDBR group.
